# 

**Published:** 1888-12

**Authors:** 


					﻿DECEMBER, 1888.
OLD SERIES
Vol. XXV
NEW SERIES (
Vol. V.-No. 10.


WeDIGAL^WMs
JOURNAL!



W.F.WESTMORELAND.M Dj
H.V.M.MILLER.M.D.LL.D.
WuBLISHEDBY JAMES P.HARR1S0N&.CK
<  ____________Atlanta ,ca. Ja
suMomrrioNi n.so a year.
				

